# CD4+ T-Cell Epitope Prediction by Combined Analysis of Antigen Conformational Flexibility and Peptide-MHCII Binding Affinity

**DOI:** 10.1021/acs.biochem.2c00237

**Published:** 2022-07-14

**Authors:** Tysheena Charles, Daniel L. Moss, Pawan Bhat, Peyton W. Moore, Nicholas A. Kummer, Avik Bhattacharya, Samuel J. Landry, Ramgopal R. Mettu

**Affiliations:** † Department of Biochemistry and Molecular Biology, Tulane University School of Medicine, New Orleans, Louisiana 70112, United States; ‡ Department of Computer Science, Tulane University, New Orleans, Louisiana 70118, United States

## Abstract

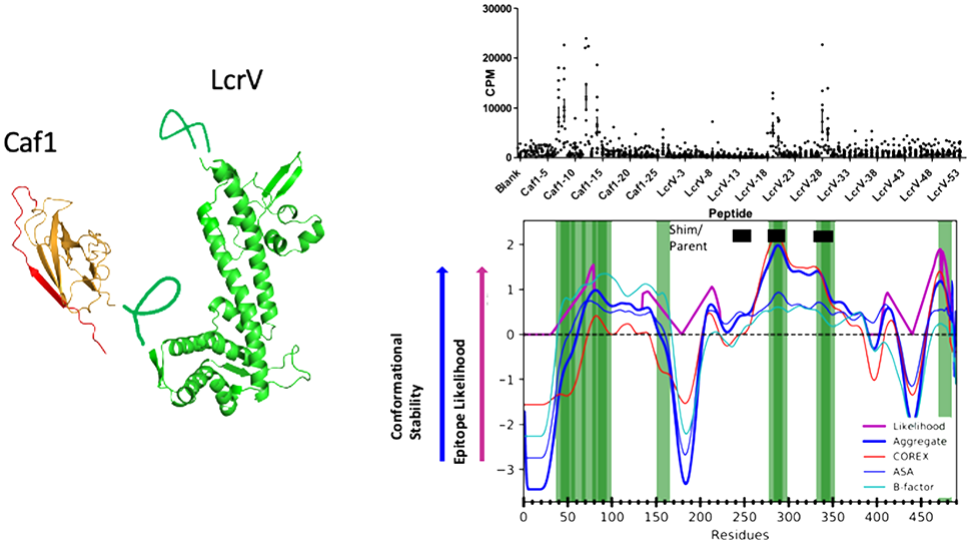

Antigen processing in the class II MHC pathway depends on conventional proteolytic enzymes, potentially acting on antigens in native-like conformational states. CD4+ epitope dominance arises from a competition among antigen folding, proteolysis, and MHCII binding. Protease-sensitive sites, linear antibody epitopes, and CD4+ T-cell epitopes were mapped in plague vaccine candidate F1-V to evaluate the various contributions to CD4+ epitope dominance. Using X-ray crystal structures, antigen processing likelihood (APL) predicts CD4+ epitopes with significant accuracy for F1-V without considering peptide-MHCII binding affinity. We also show that APL achieves excellent performance over two benchmark antigen sets. The profiles of conformational flexibility derived from the X-ray crystal structures of the F1-V proteins, Caf1 and LcrV, were similar to the biochemical profiles of linear antibody epitope reactivity and protease sensitivity, suggesting that the role of structure in proteolysis was captured by the analysis of the crystal structures. The patterns of CD4+ T-cell epitope dominance in C57BL/6, CBA, and BALB/c mice were compared to epitope predictions based on APL, MHCII binding, or both. For a sample of 13 diverse antigens, the accuracy of epitope prediction by the combination of APL and I-A^b^-MHCII-peptide affinity reached 36%. When MHCII allele specificity was also diverse, such as in human immunity, prediction of dominant epitopes by APL alone reached 42% when using a stringent scoring threshold. Because dominant CD4+ epitopes tend to occur in conformationally stable antigen domains, crystal structures typically are available for analysis by APL, and thus, the requirement for a crystal structure is not a severe limitation.

Rational vaccine design continues to be challenging, due in no small part to the multiple disparate mechanisms and signals that regulate the strength and specificity of the immune response. Antibodies and T cells form the core of adaptive immune responses, but they depend on each other and on potent signals from the innate immune system.^1^ T cells recognize polypeptide fragments displayed on the cell surface by polymorphic major histocompatibility complex (MHC) molecules. The two main types of MHC molecules, class I and class II, differ in their source of peptides and the type of T cells that recognize them.^2,3^ Class I MHC molecules (MHCI) present mostly endogenous peptides and are recognized by CD8+ T cells. Class II MHC molecules (MHCII) present mostly exogenous peptides and are recognized by CD4+ T cells.

The analysis of large numbers of natural and synthetic MHC-bound peptides, combined with the study of X-ray crystal structures, revealed that the specificity of peptide binding to MHCI and MHCII can be explained by the shape and chemical environment of the peptide binding cleft.^4−6^ A substantial degree of variability in peptide specificity derives from the polymorphism of MHCI and MHCII molecules, wherein variant residues in the peptide binding cleft modulate peptide binding specificity. In addition, the individual MHCII molecules are substantially more permissive in peptide specificity than their MHCI counterparts. Whereas peptide binding to MHCI depends more on contacts with peptide side chains, peptide binding to MHCII depends on hydrogen bonds to the peptide backbone.^7^ Whereas the MHCI cleft is deep and terminates in closed ends that completely bury the peptide termini, the MHCII cleft is shallow and terminates in open ends that allow the same peptide to bind in multiple different registers. The existence of multiple registers of peptide binding can explain how a single peptide sequence can give rise to multiple distinct epitopes, and the different registers are thought to be selected by different circumstances of binding.^8,9^ Peptide binding is also influenced by the regulated activity of the peptide-MHCII-exchange catalyst DM.^10^

In general, the MHCI and MHCII display antigens that have been processed in the cytoplasm and endolysosome, respectively.^3^ For MHCI, antigens are targeted for degradation by the ubiquitin proteasome pathway. The antigens are tagged with ubiquitin, unfolded by the ATP-dependent 19S regulatory cap, and then threaded into the proteasome core for degradation. Peptides released from the proteasome are transported by TAP into the endoplasmic reticulum, where they assemble with MHCI during folding. For MHCII, antigens are internalized by pinocytosis, receptor-mediated endocytosis, or phagocytosis and proteolyzed by conventional proteases in the endolysosome at a moderately low pH. Then the proteolytic fragments bind to MHCII, which simultaneously becomes available by the proteolytic processing of its dedicated chaperone, the invariant chain. One notable protein unfolding activity in the MHCII pathway is the γ-interferon inducible lysosomal thioreductase (GILT). In the absence of GILT, disulfide bonds reshape CD4+ epitope dominance patterns and can severely reduce immunogenicity.^12,13^ Major distinctions for the MHCII pathway are the lack of separation between antigen processing and peptide-MHCII binding and the lack of an ATP-dependent unfolding activity in the endolysosome, other than acidification.

Current CD4+ epitope prediction tools are based on the binding affinity of the peptide for the MHCII molecule.^4^ The variability in peptide length, weak sequence dependence of peptide binding, and potential for multiple binding registers cause difficulty in predicting MHCII peptide ligands. The potential for proteolytic mechanisms to limit the availability of MHCII ligands has long been recognized.^14,15^ Recent studies have identified sequence signatures near the ends of MHCII ligands eluted from MHCII complexes.^16,17^ These strategies have yielded modest improvements in the prediction of MHCII ligands. Although the accuracy measured by receiver–operator characteristic (ROC) curves for MHCII binding reaches 0.75, the prediction of actual T-cell responses has been daunting. When a small, high-scoring fraction of peptides has been tested for restimulation of T-cell responses, a very low hit rate (<20%) has been observed.^18−21^

Numerous studies have documented a role for antigen conformational stability in antigen processing and epitope presentation.^21−26^ Studies from this lab have shown that CD4+ T-cell epitopes are found adjacent to flexible regions of the antigen.^13,27−29^ These studies led to a generalized model explaining the importance of antigen structure in CD4+ epitope immunodominance.^27^ In this model, the antigen is proteolyzed within the flexible loops, which allows intervening segments to be separated from the rest of the protein upon binding to the MHCII molecule. The protein segments that are bound to the MHCII molecule continue to be protected while the termini are trimmed by further proteolysis, and then the peptide-MHC complexes are transported to the surface of the cell.

We selected a bacterial subunit vaccine as a model antigen for a study of CD4+ epitope prediction using conformational stability and MHCII binding affinity. Antibodies are crucial for protection against infection by *Yersinia pestis*, the causative agent of plague,^30^ and antibodies in turn depend on CD4+ T cells for the signals that promote B-cell class switching and affinity maturation. Because the antibodies generally target extracellular proteins, vaccine development has focused on whole organisms and on cellular fractions that include the envelope, cell wall, capsule, and secreted protein subunits.^31^ Two proteins that have advanced in plague vaccine research are capsular protein Caf1 and type III secretion component LcrV. In an effort to maximize the protectiveness of a single vaccine, genes for Caf1 and LcrV have been fused to produce a single recombinant protein, F1-V.^32^ Although protective in mice, protection in non-human primates was inadequate, and more advanced vaccine candidates are being developed.^33^ CD4+ T-cell epitopes for both LcrV and Caf1 have been mapped in mice that had been immunized with the recombinant protein or peptides in multiple mouse strains and using various vaccine formulations.^34−36^ In the case of Caf1, the efficiency of CD4+ epitope presentation to T-cell hybridomas correlated with availability in the folded structure.^37^

Here we have analyzed the potential for native structure in F1-V to shape the pathways of antigen processing, as modeled by F1-V fragmentation in limited proteolysis. Fragmentation was consistent with the accessibility of cleavage sites predicted from the X-ray crystal structures of Caf1 and LcrV and with the accessibility to antibodies against linear epitopes. MHCII binding and structure-based methods for predicting the CD4+ epitopes of F1-V were evaluated by comparison to epitope maps obtained in three strains of mice. The most accurate epitope prediction arose from a combination of methods that took into account MHC II binding and structure-based limitations on antigen processing.

## Experimental Procedures

### Proteins and Peptides

Recombinant subunit vaccines LcrV (also known as V-antigen) and F1-V were obtained from Biodefense and Emerging Infections Research Resources Repositiory (*BEI*resources). The 17-mer peptides spanning the entire F1-V were also obtained from *BEI*resources. The peptides spanned Caf1 in six-residue steps and overlapped by 11 residues and spanned LcrV in five-residue steps and overlapped by 12 residues. Peptides were dissolved in 1 mL of dimethyl sulfoxide (DMSO), 0.05% trifluoroacetic acid, 70% acetonitrile, or 6 M guanidine-HCl as recommended by the supplier to afford a final concentration of 1 mg/mL.

### Acid-Induced Denaturation of LcrV

For acid-induced unfolding experiments, the hydrophobic dye BIS-ANS (4,4′-dianilino-1,1′-binaphthyl-5,5′-disulfonic acid, Invitrogen) was used to monitor protein unfolding by fluorescence spectroscopy with an excitation wavelength of 390 nm. Emission was scanned from 400 to 500 nm with a Photon Technology International Fluorescence Spectrometer. Different pH conditions were generated using phosphate-citrate buffer, where 0.2 M dibasic sodium phosphate and 0.1 M citric acid were mixed until the desired pH was reached. Protein was mixed with dye in phosphate-citrate buffer ranging from pH 7.6 to 2.6 at concentrations of 0.1 μM protein and 1.0 μM dye. Fluorescence intensities averaged for the range of 476–485 nm and for three replicates were analyzed by nonlinear regression using a sigmoidal dose–response variable slope regression model (Prism 6).

### Limited Proteolysis

Proteolysis experiments were conducted in phosphate-citrate buffer at the indicated pH and formulated as described above. After incubation, all proteolysis reactions were terminated by the addition of an equal volume of Bio-Rad Laemmli sample buffer containing 1 mM phenylmethanesulfonyl fluoride (PMSF) and 150 mM 2-mercaptoethanol. Samples were boiled for 5 min and then analyzed by sodium dodecyl sulfate–polyacrylamide gel electrophoresis (SDS–PAGE), using a 4% to 20% Bio-Rad TGX gradient gel. Gels were stained with Coomassie blue and scanned on a Bio-Rad Chemidoc MP imaging system and analyzed with Bio-Rad ImageLab software. Trypsin-limited proteolysis experiments were performed in a volume of 20 μL with 5 μg of F1-V protein and 0, 1.25, or 2.50 μg of trypsin for 30 min at 25 °C. Elastase-limited proteolysis experiments were performed in 300 μL of phosphate-citrate buffer (pH 7.6) with 90 μg of F1-V protein and 0.9 μg of porcine elastase (Millipore-Sigma). The reaction mixture was incubated at 37 °C for 1 h with 20 μL aliquots taken every 5 min, dispensed into microcentrifuge tubes containing loading buffer, boiled, and analyzed as described above. Cathespin S-limited proteolysis experiments were performed in 40 μL of phosphate-citrate buffer (pH 7.6) containing 18 μg of F1-V protein or LcrV protein, 5 mM dithiothreitol, and 0.5 μg of cathepsin S (Milipore-Sigma). The reaction mixture was incubated at 37 °C for 30 min with 5 μL aliquots taken every 5 min. Aliquots were prepared for and analyzed by SDS–PAGE as described above.

### Mass Spectrometry of Limited Proteolysis Fragments

Proteolytic cleavage sites were identified by trypsin sequencing of fragments excised from SDS–PAGE gels. Briefly, each gel slice was destained twice using a 20-volume excess of 50 mM ammonium bicarbonate and 50% methanol for 20 min. Destained gel slices were dehydrated by incubation in a 20-volume excess of 75% acetonitrile for 20 min. Dried slices were then incubated in a 5-volume excess of 20 μg/mL mass spectrometry-grade trypsin dissolved in 50 mM ammonium bicarbonate at 37 °C overnight. Each sample was subjected to a 60 min chromatographic method employing a gradient from 2% to 25% acetonitrile in 0.1% formic acid (ACN/FA) over the course of 30 min, a gradient to 50% ACN/FA for an additional 10 min, a step to 90% ACN/FA for 8 min, and a re-equilibration into 2% ACN/FA. Chromatography was carried out in a trap-and-load format using a PicoChip source (New Objective, Woburn, MA); the trap column was a C18 PepMap 100, 5 μm, 100 Å column, and the separation column was a PicoChip REPROSIL-Pur C18-AQ, 3 μm, 120 Å, 105 mm column. The entire run was performed with a flow rate of 0.3 μL/min. Survey scans were performed in the Orbitrap utilizing a resolution of 120 000 between *m*/*z* 375 and 1600. Data-dependent MS2 scans were performed in the linear ion trap using a collision-induced dissociation (CID) of 25%. Raw data were searched using Proteome Discoverer 2.2 using SEQUEST. The Protein FASTA database was *Mus musculus* (TaxID = 10090) version 2017-07-05 with the PE-III sequence added. Static modifications included carbamidomethyl on cysteines (57.021) and dynamic modification of oxidation of methionine (15.9949). The parent ion tolerance was 10 ppm; the fragment mass tolerance was 0.6 Da, and the maximum number of missed cleavages was set to 2. Only high-scoring peptides were considered utilizing a false discovery rate (FDR) of 1%.

### Western Blots of Proteolysis Experiments

After limited proteolysis of F1-V with trypsin, SDS–PAGE gels were run as described. Following electrophoresis, gels were transferred to a polyvinylidene difluoride membrane using the Bio-Rad transblot turbo transfer system and packs. Membranes were briefly stained with ponceau (Milipore-Sigma) and imaged. Membranes were then blocked for 1 h at room temperature with phosphate-buffered saline and 0.05% Tween 20 (PBST) containing 2.5% nonfat dry milk (NFDM). Membranes were rinsed once with PBST and incubated overnight at 4 °C with a polyclonal goat antiserum specific for F1-V or LcrV (*BEI*resources, NR-31024 and NR-31022) diluted 1:10000 in PBST with 2.5% NFDM. The following day blots were washed three times with PBST for 5 min at room temperature followed by a 1 h incubation with the donkey anti-goat Alexa Flour 488-conjugated secondary antibody (Invitrogen) at a 1:10000 dilution in PBST with 2.5% NFDM in the dark. Blots were washed three times for 5 min with PBST and imaged on a Bio-Rad Chemidoc MP imaging system.

### Immunization

Groups of ten 6–8-week-old female C57BL/6 and CBA/J mice were obtained from Jackson Laboratories, and ten 6–8-week-old BALB/c mice were obtained from Charles River Laboratories, Inc. The mice were immunized intranasally with 10 μg of recombinant subunit vaccine, F1-V, and 5 μg of mutant (R192G) heat-labile toxin (mLT) as an adjuvant in a final volume of 10 μL. During immunizations, mice were anesthetized using isoflurane in O_2_ within an induction chamber. Intranasal administration was delivered by pipetting 5 μL into each nostril. The mice received two boosts of the same mixture at 2 week intervals. Mice were sacrificed via CO_2_ asphyxiation 1 week after the final boost. Spleens and cardiac blood were obtained from each mouse and processed further. All mouse experiments followed institutional guidelines approved by the Tulane Animal Care and Use Committee.

### Isolation of Mouse Serum

Immediately following CO_2_ asphyxiation, cardiac blood was drawn using a 1 mL syringe with a 21 gauge needle. Whole blood was placed in a BD Microtainer gold serum tube (BD Biosciences) for at least 1 h before processing. To collect serum, tubes were centrifuged for 90 s at 13 000 rpm in a tabletop centrifuge. The serum from each mouse was transferred into sterile 1.5 mL microcentrifuge tubes and stored at −20 °C.

### Mapping of Linear B-Cell Epitopes

A single peptide at a concentration of 4 μg/mL, in 0.1 M sodium bicarbonate, was added to each well of a flat-bottom 96-well plate and placed at 4 °C overnight. The next morning, the plates were washed five times with ELISA wash buffer (10% Triton-X in PBS), using an ELISA plate washer, and blocked in PBS containing 200 μL of 0.5% Tween 20, 4% whey, and 10% fetal bovine serum (PBS/T/W + 10% FBS) for 30 min at room temperature. Blocking buffer was removed, and the plate was washed. The mouse serum (primary antibody) was diluted in PBS/T/W + 10% FBS to a final concentration of 1:100, and 100 μL was placed on the plate for 1 h at room temperature. After 1 h, the plates were washed and 100 μL of a secondary antibody, horseradish peroxidase (HRP) goat anti-mouse IgG (Zymed), was added at a concentration of 1:2000 in PBS/T/W + 10% FBS. The secondary antibody was detected by addition of a developing solution [0.02% 3,3′,5,5′-tetramethylbenzidine (TMB) and 0.01% hydrogen peroxide in 0.1 M sodium acetate buffer (pH 6.0)] and allowing the color to develop for 3 min. The reaction was stopped by addition of 1 M phosphoric acid, and the absorbance was measured at 450 nm.

### T-Cell Proliferation

Spleens were harvested and placed in cold C tubes (MiltenyiBiotec) containing 3 mL of 10 mM phosphate-buffered saline (PBS) (pH 7.2), 0.5% bovine serum albumin (BSA), and 2 mM EDTA. Tubes remained on ice until the spleens were homogenized using the GentleMACSTMDissociator (MiltenyiBiotec). Homogenized spleens were poured through a 40 μm cell strainer (Fisher Biosciences), and the dissociated cells were collected in a 50 mL conical tube. The cell strainer was washed with 5 mL of PBS (pH 7.2), 0.5% BSA, and 2 mM EDTA to remove any cells that were attached to the mesh screen. Cells were pelleted by centrifuging the tubes at 500*g* for 7 min. Red blood cells (RBC) were lysed by addition of 1 mL of RBC lysing buffer (Sigma) and mixing for 2.5 min. The reaction was stopped by addition of 20 mL of RPMI 1640 medium to each tube. Splenocytes were pelleted by centrifugation at 500*g* for 7 min. The supernatant was decanted, and the cells were resuspended in 5 mL of complete medium (RPMI 1640 with 2 mM l-glutamine, 10% FBS, 100 units/mL penicillin, and 100 μg/mL streptomycin). The cell count was determined by adding 5 μL of cells to 95 μL of 0.4% trypan blue and using 10 μL of that solution to be counted by the Countess Automated Cell Counter (Invitrogen).

For the proliferation assay, splenocytes were plated in a Corning Costar 96-well round-bottom cell culture plate (Sigma-Aldrich) at a cell density of 2.5 × 10^5^ cells in a final volume of 170 μL. Peptides and positive controls (F1-V recombinant protein) were added in a volume of 30 μL at levels of 0.4 and 2 μg/well, respectively. Plates were incubated at 37 °C in a 5% CO_2_ environment for 72 h. After 72 h, 1.0 μCi of [^3^H]thymidine was added to each well. Cultures were incubated for an additional 18 h before the cells were harvested onto a glass filter mat (Skatron) using a cell harvester.

Filters were placed in a 20 mL scintillation vial and allowed to dry for 24 h. The next day, 10 mL of Opti-Fluor O (PerkinElmer) scintillation fluid was added to each vial and cell proliferation was determined by measuring the levels of [^3^H]thymidine incorporation using a scintillation counter. The response of a single mouse was considered positive if the stimulation index (SI) was >2, which corresponded to two standard deviations above the average proliferation of unstimulated cultures. Peptides were considered immunodominant if six or more mice responded.

### Prediction of MHCII Affinity

Peptides having high affinity for the MHCII molecules in each mouse strain were identified using the NetMHCII 2.3 Server.^4^ This server makes use of a neural network-based approach that is trained on a large data set of more than 14 000 quantitative peptide MHC binding values that cover 14 alleles. Due to the importance of the peptide flanking residues, each peptide encoded into the method includes information about the peptide binding core and the length and composition of the residues flanking the core. The sequences for the V antigen and F1 were added to the web-based server, and the IC_50_ values of the 17-mers with I-A^b^, I-A^k^, and I-A^d^ were retrieved.

### Antigen Processing Likelihood

**Figure 1:**
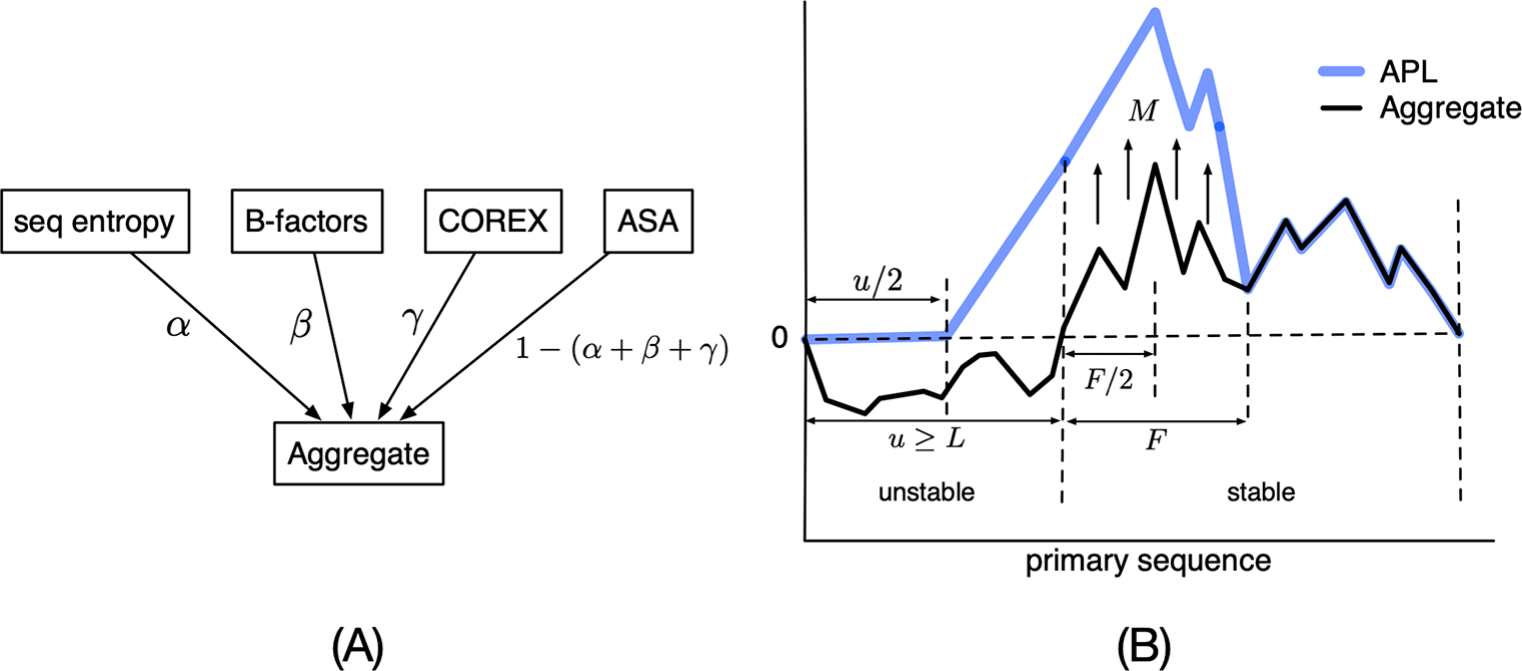
Our algorithm for computing antigen processing likelihood with associated parameters. (A) Parameters used to combine data sources. (B) Schematic of the weighting scheme and associated parameters in our algorithm.

To predict antigen processing likelihood (APL), we use an updated version of the algorithm described in ref (38). Our current algorithm works by first aggregating input sources of conformational stability data. We typically use four sources of data: sequence entropy, crystallographic *b*-factors, COREX score, and solvent-accessible surface area. The aggregation procedure works by computing a weighted combination of *z*-scores computed from the given sources of data (Figure 1A). Thus, other sources of data as well as a different number of sources can be used as input; we can also vary the contribution of each source of data based on its estimated importance. In the resulting stability profile, we consider residues having a positive *z*-score as “stable” and all other residues as “unstable”. Computing APL proceeds by upweighting all regions in which the stability profile undergoes a transition from stable to unstable regions (or vice versa). Upweighting at these regions of transitional stability attempts to capture the increased likelihood of proteolysis, which in turn would capture the corresponding increased likelihood of epitopes adjacent to proteolytic sites.

In this paper, we follow the upweighting procedure described in ref (38), but with generalized constants that are optimized (Figure 1B). For a given transitional region with adjacent stable and unstable components, the upweighting scheme scales up the weights of the *F* residues in the “flank” closest to the transition (in the stable component) by a magnification factor *M*. The weights of *U*/2 residues closest to the transition (in the unstable component) are set in a linearly increasing fashion, starting from the midpoint of the unstable component and ending at the midpoint of the upweighted flank.

### Parameter Optimization

We seek to optimize the parameters used in the algorithm to achieve a maximum positive predictive value (PPV) that is calculated as the ratio of true positives to identified positives (i.e., true positives plus false positives). We optimize parameters for each antigen of interest (the “test antigen”) by utilizing a methodology analogous to the standard cross-fold validation methodology from machine learning. The parameters we consider are the individual weights of the input data sources (i.e., “input-source weights” defined by α, β, and γ) and the parameters of the upweighting procedure (i.e., “algorithm parameters”) that define the allowable loop size (*L*), magnification factor (*M*), and portions of stable region (*F*) that are upweighted. The range considered for each input-source weight was between 0 and 1, in increments of 0.1 with the constraint that they sum to 1. The ranges for algorithm parameters were as follows. Loop sizes of 0–30 were considered in increments of five residues, and flank sizes of 9–30 were considered in increments of five residues. Finally, when applicable, the ratio of APL to MHC scores (i.e., for single-allele data sets) is also searched in the range of 0–1 in increments of 0.1. This ratio is used to generate the combined scoring scheme shown in Tables 1 and 2.

**Table 1:** Accuracy of Prediction Methods for F1-V CD4+ Epitopes

	hits for seven peptides predicted at the 90th percentile and ROC-AUC	
prediction method	C57BL/6	CBA
APL	2/0.72^a^	1/0.69^a^
MHCII	2/0.76^b^	2/0.53
APL and MHCII	2/0.82^c^	1/0.62
APL (with F-LE)	2/0.73^a^	3/0.69^a^
APL (with F-LE) and MHCII	4/0.81^c^	2/0.64
APL (with proteolysis)	2/0.69^a^	3/0.69^a^
APL (with proteolysis) and MHCII	4/0.79^b^	3/0.63
APL (F-LE only)	2/0.57	1/0.64
APL (proteolysis only)	3/0.63	1/0.73^a^
APL (F-LE and proteolysis)	0/0.56	3/0.75^b^
APL (F-LE and proteolysis) and MHCII	3/0.71^a^	3/0.69^a^

a *p* < 0.05.

b *p* < 0.01.

c *p* < 0.001.

**Table 2:** Numbers of Peptides: Epitopes, High-Scoring, and Correctly Predicted for C57BL/6 Mice

antigen	no. of epitopes	ref	PDB entry	total no. of peptides	threshold (no. of peptides)	APL	MHCII binding	combined
HIV gp120	5	(13)	3JWO	46	0.10 (4)	0	1	1
plague F1-V	12	this work	1P5V, 4JBU	78	0.09 (7)	2	2	2
*M.t*. Ag85A	5	(42)	1DQZ^a^	27	0.09 (2)	0	0	0
friend V. Env	6	(43)	1AOL	37	0.09 (3)	1	2	2
GFP	1	(44)	2QLE	17	0.10 (1)	1	0	1
*M.t.* Mpt51	1	(45)	1R88	25	0.10 (2)	0	0	0
*M.t.* PstS	3	(46)	1PC3	32	0.10 (3)	0	1	1
VSV-G	2	(47)	2CMZ	44	0.10 (4)	0	1	1
OVA	9	(48)	1OVA	75	0.10 (7)	0	3	3
flu HA	9	(49)	3LZG	83	0.09 (7)	2	1	2
flu NA	9	(50)	3CYE	63	0.09 (5)	1	2	2
LLO	3	(51)	4CDB	48	0.10 (4)	1	1	1
TMEV VP2	1	(52)	1TME	25	0.10 (2)	0	1	1
average	4.8			46	0.10 (3.92)	0.62	1.15	1.31

a Homology model template.

We optimize the parameters for each test antigen individually, in two stages. We refer to the remaining antigens as the “training set”. First, we perform an optimization for the algorithm parameters on all antigens in the training set (from either C57/BL6 mice or human subjects). We then fix these algorithm parameters and optimize the input-source weights in the training set. For the final input-source weights, we take the average relative weights of the four sources, yielding the top 10% PPV values. We then conduct a final round with these input-source weights to reoptimize the algorithm parameters to obtain the final choice for the test antigen.

### Statistical Tests

The Wilcoxon signed rank test for identification of a positive T-cell response, ROC-AUC with significance for prediction accuracy, one-way analysis of variance (ANOVA) with repeated measures, and Tukey multiple comparisons for hit frequency of prediction methods versus “random” selection of mouse epitopes (epitope frequency), a paired *t* test for APL versus “random” selection of human epitopes, and a paired *t* test for the number of hits predicted by combined versus MHCII were calculated using GraphPad Prism.

## Results

### Acid-Induced Denaturation of LcrV

**Figure 2:**
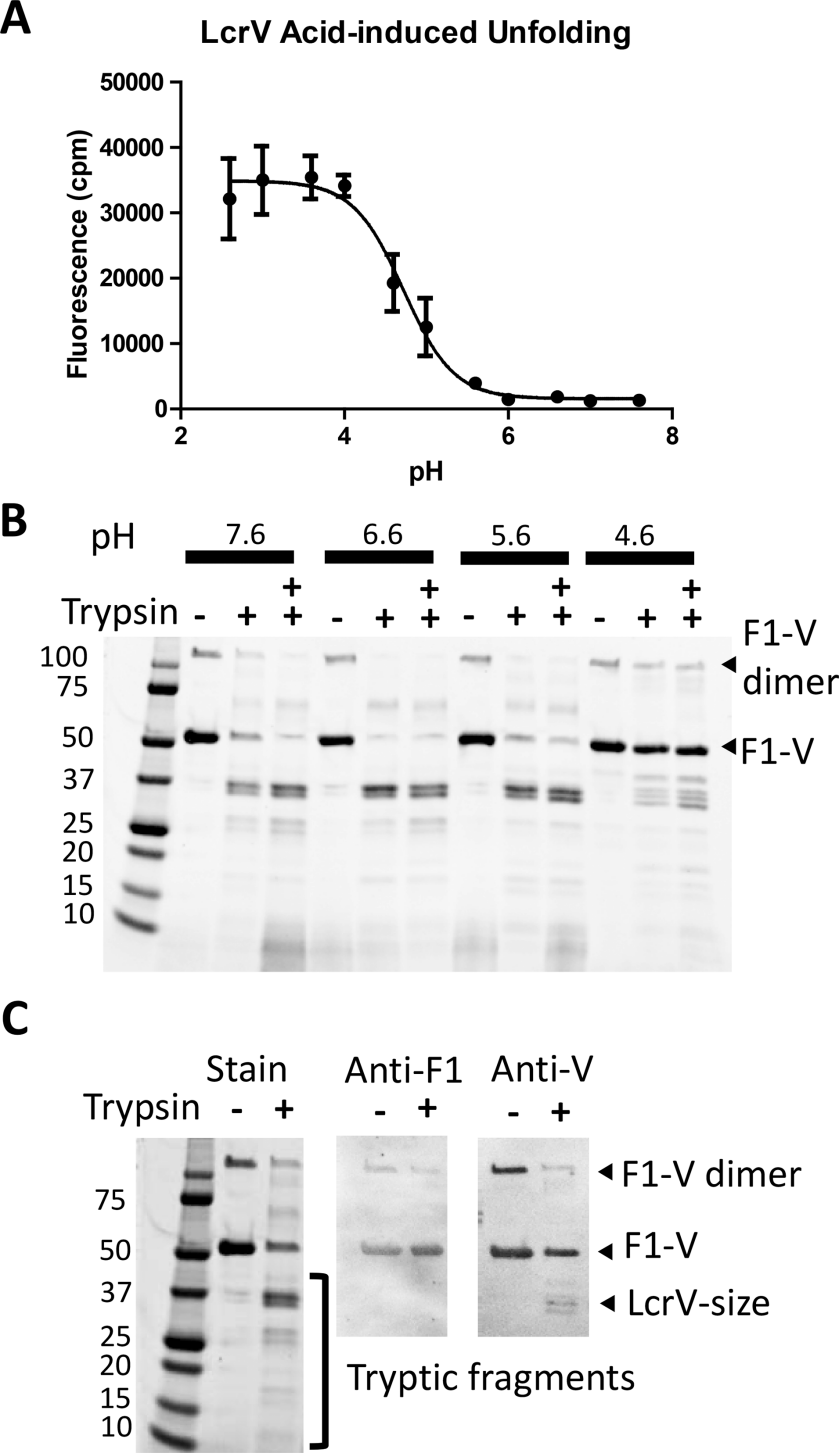
Conformational stability and acid resistance in LcrV and F1-V. (A) LcrV resists acid-induced denaturation down to at least pH 5.6, as reported by minimal binding of the fluorescent dye bis-ANS. (B) According to SDS–PAGE and Coomassie staining, the fragmentation pattern from limited proteolysis with trypsin remains consistent at pH 7.6, 6.6, and 5.6, suggesting that the folded structure in F1-V resists proteolysis down to pH 5.6. Two pluses indicate doubling of the trypsin concentration. Similar results were obtained for limited proteolysis with proteinase K (Figure S1). (C) In Western blots, prominent 37 kDa fragments from limited proteolysis with trypsin are decorated by anti-LcrV (anti-V) antibodies and thus correspond to LcrV. *BEI*resources depletes the dimeric form of F1-V from the preparation, but an SDS-resistant F1-V dimer constitutes approximately 25% of the mass. The immunological properties of oligomeric and monomeric forms of F1-V were reported to be indistinguishable.^39^

We hypothesized that the conformation of LcrV would remain native-like under the moderately acidic conditions of the antigen-processing compartment;^40^ thus, the fragmentation of LcrV would be modulated by structure, rather than any primary sequence specificity of the protease. LcrV was expected to undergo a cooperative unfolding transition as the buffer pH became progressively lower. Acid-induced denaturation of LcrV was monitored by the increase in 4,4′-dianilino-1,1′-binaphthyl-5,5′-disulfonic acid (Bis-ANS) fluorescence, resulting from its increased level of binding to the denatured protein. The fluorescence was measured with three replicates at each pH. The average fluorescence was calculated from 476 to 485 nm for each replicate, and then the average and standard deviation were calculated for all replicates at each pH. These values were plotted in an unfolding curve (Figure 2A). The low level of bis-ANS fluorescence down to pH 5.6 indicates that F1-V remained at least 92% folded. As the pH was further decreased, LcrV unfolded with the midpoint of the transition at pH 4.7. Limited proteolysis of F1-V with trypsin and proteinase K yielded consistent fragmentation patterns at pH values as low as 5.6 (Figure 2B and Figure S1).

### Limited Proteolysis of F1-V

We hypothesized that the proteolytic fragmentation of F1-V is primarily controlled by the ability of F1-V segments to conform to the protease active site. Thus, fragmentation patterns from limited proteolysis were expected to reflect the F1-V domain structure and profile of conformational flexibility and to be somewhat insensitive to the particular protease. For example, 51 kDa F1-V has 56 potential cleavage sites for trypsin, based on the number of lysine and arginine residues. In spite of the large number of potential cleavage sites, limited proteolysis with trypsin of F1-V yielded a most prominent fragment at 37 kDa, which is the expected size of the C-terminal LcrV portion of F1-V (Figure 2C). The identity of this fragment as essentially LcrV was supported by Western blotting, which revealed the decoration of an LcrV-sized band with anti-LcrV antibodies but not with anti-Caf1 antibodies. Similar results were obtained from limited proteolysis with other proteases, including elastase, proteinase K, and cathepsin S.

**Figure 3:**
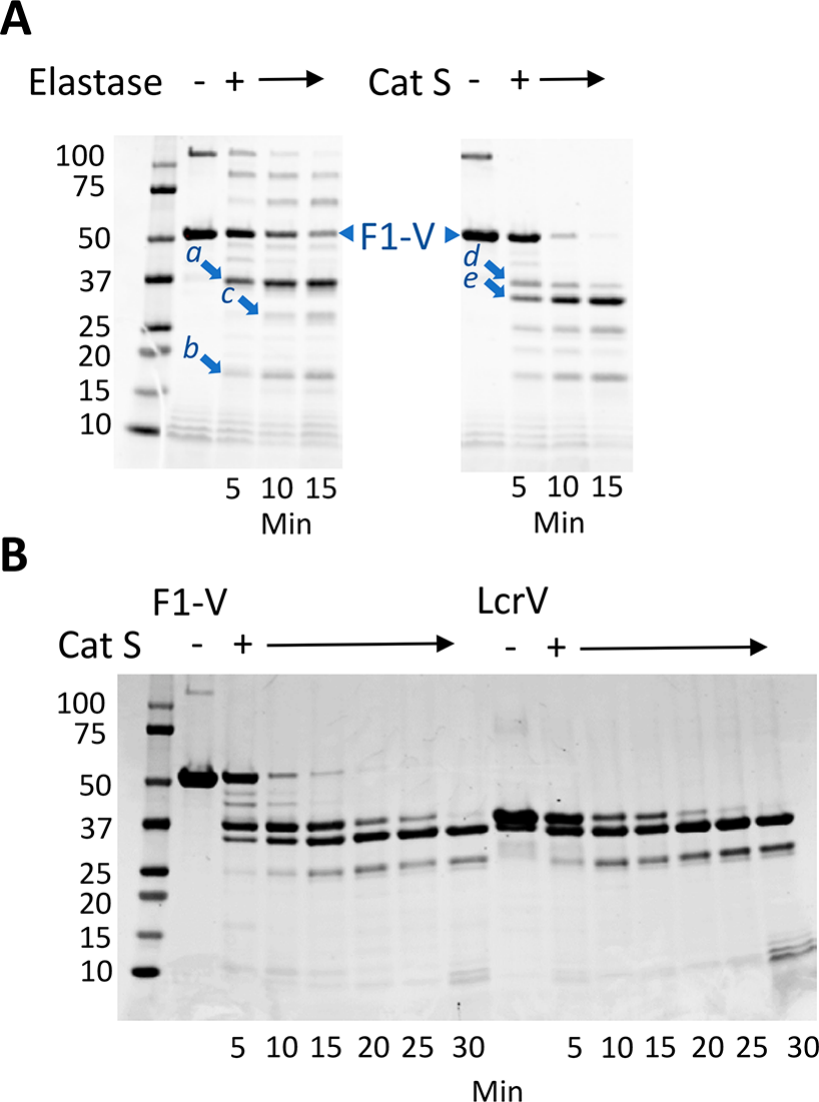
Limited proteolysis of F1-V with elastase and cathepsin S yields similar fragmentation patterns. (A) According to SDS–PAGE and Coomassie staining, digestion with elastase and cathepsin S each generates fragments of 37, 25, and 17 kDa. Cathepsin S also generates a 30 kDa fragment. Arrows indicate the fragments that were identified by tryptic proteomics to contain the following residues of F1-V: fragments a and d, 176–479; fragment b, 75–175; fragment c, 176–403; fragment e, 235–479. (B) The similarity of fragmentation patterns for digestion of F1-V and LcrV by cathepsin S confirms that the 37, 30, and 25 kDa fragments of F1-V correspond to portions of LcrV.

**Figure 4:**
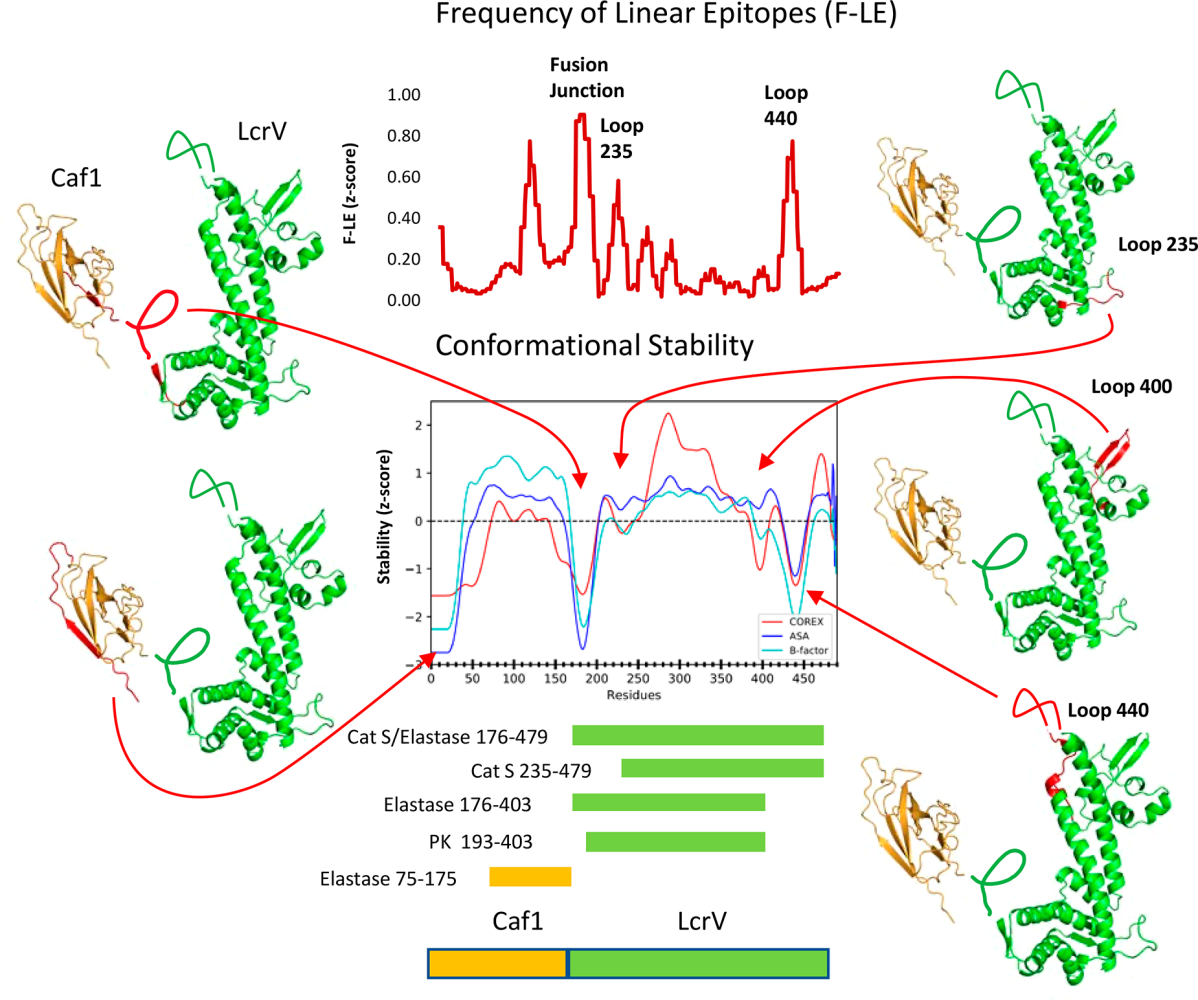
F1-V cleavage sites observed by limited proteolysis correspond to conformationally unstable, solvent-exposed segments. The graph of conformational stability illustrates *z*-score profiles generated from the X-ray crystal structures of Caf1 and LcrV (Protein Data Bank entries 1Z9S and 4JBU, respectively). Above, the graph of F-LE illustrates a *z*-score profile of antibody reactivity with linear antibody epitopes (Supplemental Table 1). Amber and green bars demarcate proteolytic fragments identified by LC-MS/MS in Caf1 and LcrV, respectively. Preferred sites of proteolytic cleavage are illustrated in the ribbon diagrams, and arrows denote their positions in the profiles of conformational stability.

To further resolve the identities of proteolytic products and locations of protease cleavage sites, six major fragments from various protease digestions were excised from the gels and identified by complete tryptic digestion followed by analysis of the peptides with liquid chromatography and mass spectrometry (LC-MS/MS). The 37 kDa fragments from digestion with elastase and cathepsin S were each found to contain tryptic peptides spanning residues 176–479 (Figures 3A and 4). This segment is approximately 20 residues smaller than expected for 37 kDa full-length LcrV (residues 171–496). Because our analysis was not optimized for detecting peptides that lack the C-terminal Lys/Arg or very small peptides (fewer than eight residues), which would be produced by cleavage in the segment of residues 479–496, we concluded that the 37 kDa fragments corresponded to essentially full-length LcrV (residues 171–496). LC-MS/MS of the approximately 25 kDa elastase fragment (not shown) and 25 kDa proteinase K fragment (Figure S1) yielded tryptic peptides that span residues 176–403 and 193–403, respectively, which are consistent with cleavage near the F1-V fusion junction and at a second site C-terminal from residue 403. The 17 kDa elastase fragment yielded tryptic peptides spanning Caf1 residues 75–175. The approximately 30 kDa cathepsin S fragment yielded tryptic peptides that span residues 235–479. The assignment of the 37, 30, and 25 kDa fragments to the LcrV portion of F1-V was confirmed by the similar fragmentation pattern obtained from digestion of LcrV by cathepsin S (Figure 3B).

### Peptide-Reactive Antibody Responses

**Figure 5:**
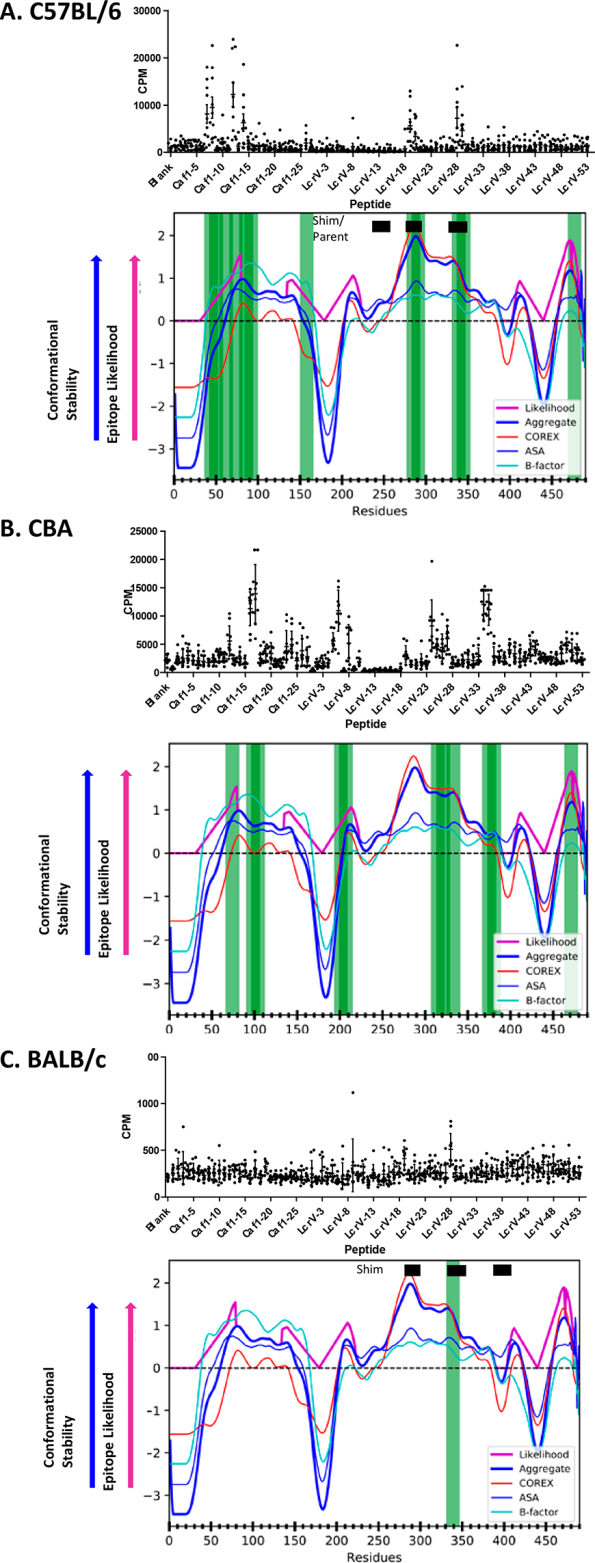
Dominant CD4+ epitopes correspond to stable segments of F1-V. CD4+ T-cell epitopes were mapped in F1-V-immunized (A) C57BL/6, (B) CBA, or (C) BALB/c mice by splenocyte stimulation with overlapping peptides and measurement of [^3^H]thymidine incorporation, detected as counts per minute (cpm). Vertical green bars indicate dominant epitope-containing peptides, which stimulated proliferation of splenocytes from at least six mice. *z*-Score profiles for various measures of conformational stability were combined into a single aggregate *z*-score. Epitope likelihood (APL) is a non-zero quantity that tracks with aggregate conformational stability and is magnified in stable segments adjacent to unstable segments (see Experimental Procedures). Horizontal black bars indicate T-cell epitopes that were mapped in earlier studies.^28,29^

Groups of 10 C57BL/6, CBA, and BALB/c mice were immunized intranasally with F1-V combined with the adjuvant, mutant heat-labile toxin (mLT) from *Escherichia coli*.^41^ Linear antibody epitopes were mapped using the antiserum of each mouse. Of 79 peptides spanning F1-V, 10 peptides reacted with antisera from a majority of mice, wherein reactivity was considered positive if the ELISA signal for a peptide exceeded that for the blank by two standard deviations. These 10 peptides occur in four clusters [peptides 18 and 19, 28–30, 35 and 36, and 70–72, corresponding to F1-V residues 103–125 (in Caf1), 171–198 (fusion junction), 212–234 (in LcrV), and 422–450 (in LcrV), respectively (Figure 5 and Supplemental Table 1)].

### Proliferative Responses

Proliferative responses to F1-V were mapped by peptide restimulation of splenocytes from mice intranasally immunized with F1-V and mLT as described above. Splenocytes from each mouse were tested with individual peptides spanning complete F1-V. For the sake of simplicity, overlapping peptides that could share a single epitope are considered two separate epitopes. Approximately 30% of the peptides were designated “positive” for each group of mice because they stimulated significant proliferation, as scored by the Wilcoxon signed rank test. A large fraction (90%, 71 peptides) were positive in at least one strain. Only a minor fraction (20%) of positive peptides was shared among two or more mouse strains.

Within the Wilcoxon-positive peptide sets, a smaller number of “dominant” peptides stimulated proliferation of splenocytes from at least six mice within a group (Figure 5 and Supplementary Table 1). For mouse strains C57BL/6 and CBA, 12 and 11 peptides, respectively, were dominant, and for BALB/c mice, only one peptide was dominant. Approximately one-fourth of the 79 peptides (22 peptides) was dominant in at least one strain. Only two peptides were dominant in multiple strains (peptides 13 and 56). Clusters of dominant epitopes were located in three regions of F1-V: the central stable region of the Caf-1 domain (residues 37–107, peptides 7–17), the central stable region of LcrV (residues 278–348, peptides 46–56), and the C-terminal stable region of LcrV (residues 464–486, peptides 77 and 78).

### Accuracy of CD4+ Epitope Prediction

Epitope mapping results were compared to CD4+ epitope predictions based on our antigen processing likelihood (APL) algorithm (outlined in Experimental Section) and peptide-MHCII binding affinity, as well as a combination of the two approaches. For APL, three measures of residue conformational flexibility and/or stability (crystallographic *b*-factor, solvent-accessible surface area, and COREX residue stability) were compiled and then input into the previously described algorithm that assigns APL to stable antigen segments adjacent to flexible antigen segments.^38^ The algorithm also accepts Shannon sequence entropy as input, but the small number of proteins homologous to Caf1 and LcrV precluded a reliable analysis of sequence entropy; thus, it was not included. For the MHCII binding analysis, the experimental peptide sequences were entered as input into the web form for the NETMHCII 2.3 Server; the appropriate H-2 locus was selected, and the resulting values of “1–log50k(aff)” were recorded.^4^

As described, we optimized the parameter values for the APL algorithm using a leave-out search. As described in Experimental Section, we perform separate optimizations when APL is used alone and when used in conjunction with MHC binding predictions in the combined method. The optimization criterion aims to maximize the positive predictive value at the empirical frequency of epitopes observed in the test set. The resulting parameters yield weights on the relative importance of input sources of data as well as for the APL weighting scheme and ratio for combining APL and MHC scores. To compensate for the effects of noise and artifacts in the optimization, we evaluate predictions for each test antigen using a parameter set that is the average over parameter sets achieving the top 10% of PPV values over nontest antigens.

For C57BL/6 data sets (Figure S2a), the average optimum input-source weights (across all test antigens) for the combined prediction were 0.13, 0.11, 0.25, and 0.50 for sequence conservation, *b*-factors, COREX, and ASA, respectively. For the algorithm parameters, we obtain an average magnification factor of 1.65 and average loop and flank sizes of 14 and 19 residues, respectively. For the optimal ratio of APL to MHC, we obtain 0.31. This is also interesting as it shows that over our set of antigens for single-allele epitope data, APL scoring makes a sizable contribution to optimizing PPV. Additionally, the optimal value of this parameter varies with antigen, ranging from 0.22 for Friend V Env to 0.43 for TMEV Vp2. For predictions made with APL alone, we optimized parameters separately (Figure S2b) to obtain input-source weights of 0.43, 0.20, 0.12, and 0.25 for sequence conservation, *b*-factors, COREX, and ASA, respectively, and algorithm parameters of 1.41, 17, and 21 for magnification, loop size, and flank size, respectively. Again, we note that each input source contributes substantially to the prediction, but due to weak overall performance, it is difficult to draw any substantive conclusions about the chosen parameters.

For data sets from human subjects (Figure S3), the average input-source weights were 0.17, 0.18, 0.51, and 0.15 for sequence conservation, *b*-factors, COREX, and ASA, respectively. We note that for these data sets, no MHCII binding predictions were utilized in the optimization. These values are similar to those obtained for the C57BL/6 data set, with COREX and ASA exchanging relative importance. For the algorithm parameters, the loop and flank sizes were 16 and 11, respectively, while the magnification was optimized to be higher at 2.4.

The accuracy of epitope prediction can be assessed in multiple ways. We consider two methods of evaluation, the positive-predictive value (PPV) at a particular threshold of prediction score and receiver–operator characteristic area under the curve (ROC-AUC). For the first method, we conduct leave-out testing and evaluate the PPV for each antigen at a threshold determined empirically. That is, for each test antigen under consideration, we optimize the parameters (see Experimental Section) and evaluate the number of epitopes identified at a scoring threshold determined by the average frequency of epitopes in the training set used for optimization. For C57BL/6 antigens, the average frequency of epitopes was 10% (Table 2). We compared the PPV achieved by APL alone, by MHC alone, and by the combined scoring methods against a baseline of selecting epitopes at random according to epitope frequency. For C57BL/6 antigens, whereas PPV for neither APL nor MHC binding was significant, the PPV for combined scoring (36%) was significant. For antigens in human subjects, we can evaluate only APL scoring predictions. APL achieved a PPV of 24%, which was not significantly greater than random selection at the corresponding empirical thresholds. It is important to note that the empirical threshold at which we chose to consider PPV is determined by the data set; we discuss the issue of threshold selection in practice below. Notably, when we loosen the threshold to be 50% lower than the empirical average for each nontest antigen (e.g., from 84th to 80th percentile), we achieve an improved PPV of 27%, and the prediction is significantly better than random selection (*p* < 0.02). Further decreasing the threshold retains significance but does not improve PPV.

**Figure 6:**
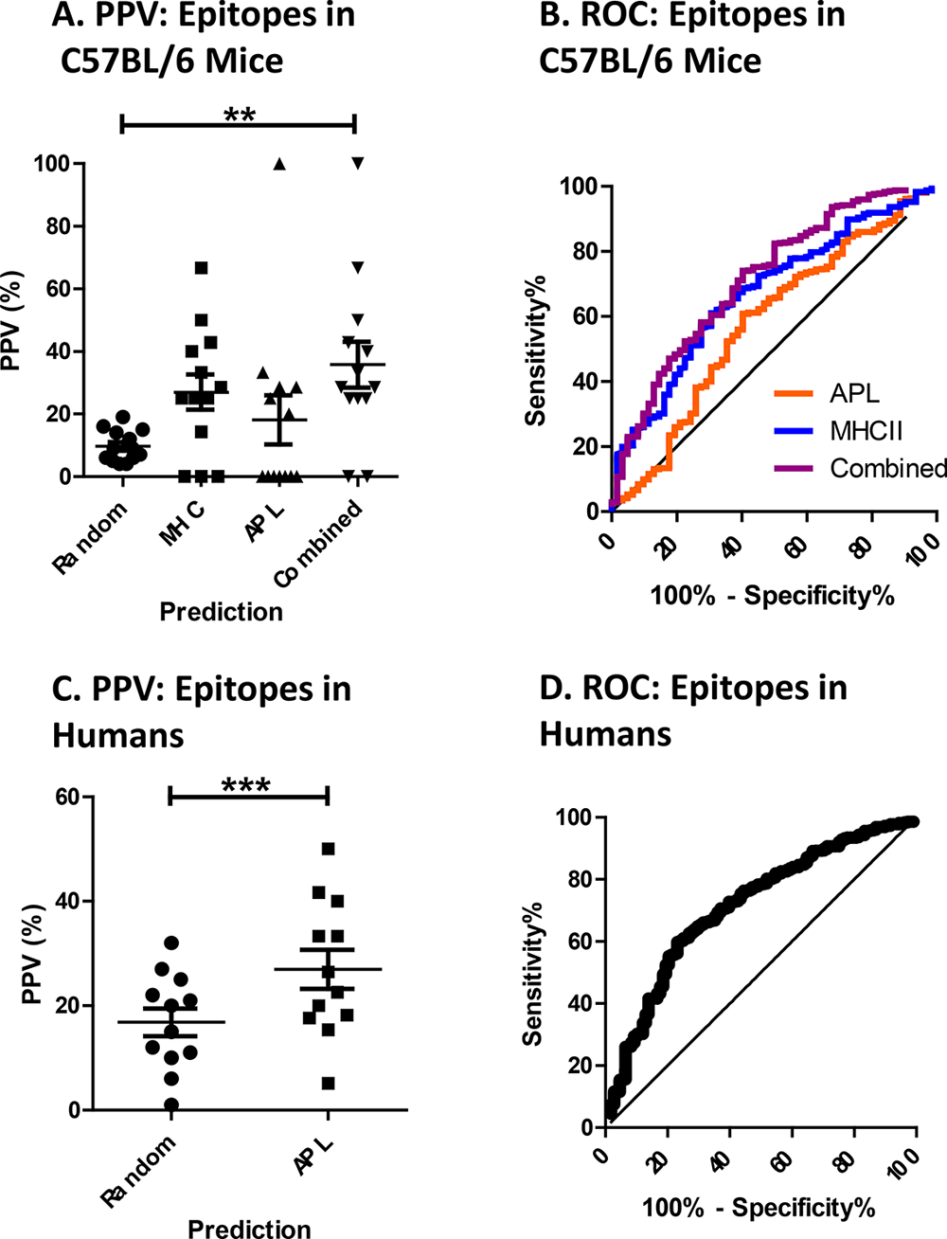
Accuracy of CD4+ epitope predictions for antigen collection. (A) Frequency of epitope hits among peptides scoring above the threshold (approximately top 10%) predicted for individual antigens using the indicated method. “Random” indicates the frequency of experimentally observed epitopes within the complete set of peptides for the antigen (i.e., frequency of epitope hits for random sampling). Asterisks indicate significance by one-way ANOVA with repeated measures and the Tukey method of multiple comparisons (*p < 0.05; **p < 0.01). (B) Accuracy for 13 antigens in C57BL/6 mice, as illustrated by the ROC curve. The indicated AUC values are significantly greater than 0.5 (*p* < 0.0001). (C) Frequency of epitope hits among peptides above the empirical threshold (approximately top 17%) scored for individual antigens using the indicated method. The asterisk indicates significance by paired *t* test (*p* < 0.05). (D) Accuracy for 12 antigens in humans, as illustrated by the ROC curve. The indicated AUC value is significantly greater than 0.5 (*p* < 0.0001).

To consider a threshold-independent performance metric, we make use of the receiver–operator characteristic (ROC). The ROC curve for each prediction can be evaluated by the area under the curve (AUC) of sensitivity versus 1 – specificity (false-positive level). If the AUC exceeds a value of 0.5 (*p* < 0.05), then the scores can be considered to have predictive power better than random. For sample sizes corresponding to the C57BL/6 and CBA epitope predictions, the ROC-AUC achieves significance at a value of approximately 0.69. Both APL binding and MHCII binding achieved significant accuracy in the prediction of dominant epitopes observed with C57BL/6 mice (Figure 6 and Table 1).

**Figure 7:**
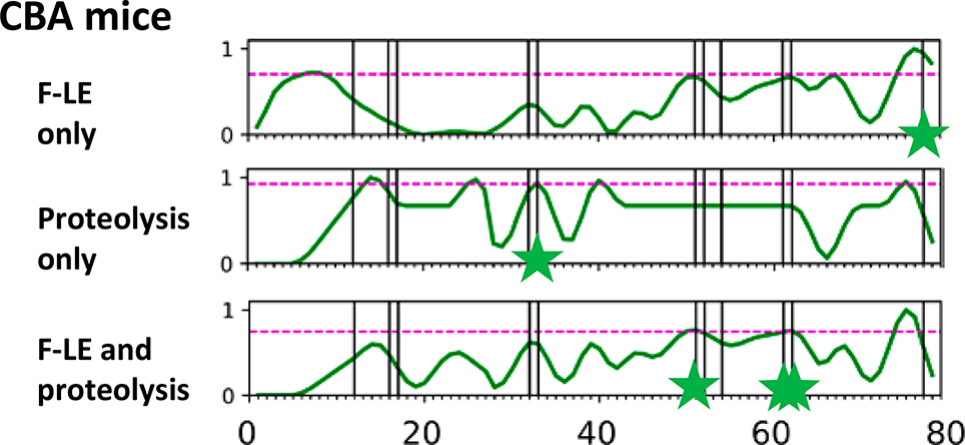
Prediction of dominant CD4+ epitopes of F1-V by APL without crystallographic structures. *z*-Score profiles generated on the basis of F-LE (Figure 5) and fragmentation by limited proteolysis (see the text) were used as input to the APL algorithm. At a threshold of 90th percentile (dashed line), APL using only these two parameters identified (stars) a significant number of the dominant F1-V epitopes in CBA mice (vertical lines).

We sought to explore improvement in APL accuracy by supplementing or replacing the crystallographic data with biochemical evidence of conformational flexibility and protease sensitivity. For the test antigen plague F1-V, the frequency of linear antibody epitopes (F-LE) was converted to a residue-by-residue *z*-score that could be included alongside the *b*-factor, solvent-accessible surface area, and COREX residue stability. Likewise, using protease sensitivity/resistance as a binary score, a value of “0” was assigned to all residues in F1-V that were excluded from proteolytic fragments and to the residues at the termini of a fragment (Figure 7). The supplementation of APL input with these profiles (all at equal weight) did not substantially change the results, suggesting that the crystallographic and biochemical approaches yield similar information about the F1-V structure with regard to epitope prediction (Table 1). Remarkably, the combination of the two biochemical parameters F-LE and protease sensitivity/resistance alone (without any crystallographic parameters) achieved significant accuracy in the prediction of F1-V CD4+ epitopes in CBA mice (Figure 7 and Table 1).

## Discussion

Protease-sensitive sites and CD4+ T-cell epitopes were mapped in F1-V to investigate the influence of antigen processing on epitope dominance. Antigen processing for the MHCII pathway occurs in an endolysosome-like compartment, where antigens, proteases, disulfide-exchange catalyst GILT, MHC II, and the MHCII-peptide-exchange catalyst DM together experience a time- and activation-dependent acidification. Because many proteins retain native-like conformations in acidic environments, we sought to test the hypothesis that the three-dimensional structure of F1-V influences CD4+ epitope dominance by limiting access to lysosomal proteases and the MHCII molecule.

We hypothesized that conformationally stable domains of F1-V could resist acidification in the antigen-processing compartment. LcrV resisted denaturation to at least pH 5.6, where 92% of the protein remained in the native structure, as reported by bis-ANS binding (Figure 2). The acid-induced unfolding of complete F1-V was not undertaken because the complex unfolding pathway of the multidomain protein would preclude analysis of the fraction unfolded. As a separate molecule, Caf1 adopts an incompletely folded metastable state prior to assembly into fibers, and its structure has been characterized only in complexes with the dedicated molecular chaperone Caf1M.^53^ An N-terminal segment of the protein must either associate with the chaperone or fold with another subunit of Caf1. Recombinant Caf1 forms a flocculant aggregate that resembles the capsule formed by *Yersinia pestis*, and denaturation reduced its protectiveness as a vaccine.^54^ Presentation of Caf1 epitopes to T-cell hybridomas was found to be increasingly dependent on antigen processing according to their position from the N-terminus to C-terminus, suggesting that the structure was progressively unraveled during processing.^37^ Our findings indicate that LcrV should be added to a list of antigens, including Caf1, Bet v 1, and *Pseudomonas aeruginosa* exotoxin domain III, for which the conformations sufficiently resist acid denaturation that they limit access to proteolytic enzymes and/or MHCII molecules.^25,37,55^

Limited proteolysis and peptide mapping of F1-V indicated that the most proteolytically sensitive sites lie near the Caf1-LcrV fusion junction. A prominent fragment of a size equal to that of LcrV (37 kDa) and that reacted with anti-LcrV antibodies was generated by each of the tested proteases (trypsin, protease K, elastase, thermolysin, and cathepsin S). Representative 37 kDa fragments from digestion with elastase and proteinase K were found by tryptic proteomics to contain the segments of residues 176–479 and 193–479, respectively (Figure 4). For comparison, under denaturing conditions, the exhaustive fragmentation of LcrV with trypsin yielded 28 peptides, none of which was larger than 4 kDa (data not shown). Likewise, a 101-residue fragment of 175-residue Caf1 was recovered from limited proteolysis with elastase, which exhibits very broad specificity and therefore is expected to cleave at many sites of an unstructured protein.^56^ Thus, the preferred cleavage of F1-V by trypsin and other proteases near the F1-V fusion junction was most likely due to conformational disorder in the region of the fusion junction, whereas the Caf1 and LcrV portions of F1-V remained relatively ordered and resistant to proteolysis.

Proteolytically sensitive sites within the LcrV portion of F1-V are associated with conformationally disordered loops that are evident in the LcrV crystal structure. A 30 kDa cathepsin S fragment of LcrV spanning residues 235–479 resulted from cleavage within a flexible loop (loop-235) that protrudes from the N-terminal globular domain of LcrV (Figure 4). Preferred C-terminal cleavage sites in F1-V were located within the C-terminal 20 residues (479–496) of LcrV or within a large unstable region of LcrV spanning residues 390–460, including hairpin loop-400 and disordered loop-440. Early fragments are likely to be subject to rapid further proteolytic cleavage, as the result of enhanced conformational flexibility in the new terminal regions. For example, we suspect that initial cleavage occurs in the large flexible loop-440 and that additional proteolytic steps shorten the polypeptide from the C-terminus to a point between residues 403 and 409, based on the tryptic proteomics.

A residue-level profile of linear antibody epitopes potentially offers an alternative source of conformational stability data that can supplement or replace crystallographic data. Antiserum reactivity with a synthetic peptide suggests that the antibody epitope is contained within the corresponding F1-V segment and that the epitope is available in the context of the native protein. To be available in the native protein, the segment must be solvent-accessible and able to conform to the binding site on the antibody.^57,58^ A majority of peptides that reacted with antibodies raised against intact F1-V corresponded to conformationally unstable antigen segments (Figure 4). The frequency of linear epitopes (F-LE) was scored as the average fraction of mice that reacted with the peptides that contain the residue. Each residue appears in three peptides, and each peptide was probed with immune serum of 10 mice from each of three mouse strains. Three clusters of the most consistently reactive peptides were in segments that are known to be disordered, one near the fusion junction and two internal loops of LcrV (loop-235 and loop-440). A fourth highly reactive cluster of peptides corresponded to a segment of Caf1 that forms a three-stranded β-sheet and had not been identified as flexible in the crystal structure. Thus, F-LE could supplement the crystallographic information in the analysis of conformationally unstable segments and possibly take the place of crystallographic information when it is unavailable.

Proliferative T-cell responses were well distributed in the F1-V sequences and punctuated by dominant epitopes that stimulated responses in a majority of mice of a given strain. For the two strains (C57BL/6 and CBA) having a single MHCII molecule (I-A^b^ and I-A^k^, respectively), approximately half of the peptides that scored positive using the Wilcoxon signed rank test stimulated a significant response in a majority of mice, and therefore are also defined as dominant epitopes. Only one peptide was dominant in the BALB/c mice. While only two of 22 dominant epitopes were shared between strains, clusters of dominant epitopes appeared to be shared among strains, e.g., spanning residues 37–107 or 278–348 (Figure 5). The clustering of non-identical epitopes could be explained by distinct but overlapping MHCII sequence preferences or by the limitation of MHCII selection to antigen segments that preferentially emerge from antigen processing.

Two of three LcrV epitopes that were previously identified in C57BL/6 mice were re-identified here, and one of three LcrV epitopes previously identified in BALB/c mice was re-identified here (Figure 5). Among the possible explanations for the differences are the use of LcrV instead of F1-V as the immunogen and different routes and adjuvants for the immunization.^34,35^ Our studies of limited proteolysis yielded no evidence that fusion to Caf1 had affected the conformation of LcrV. Thus, we favor the conclusion that the route and/or adjuvant affected antigen processing through engagement of different antigen-presenting cells or modulation of the agents of antigen processing. One striking example of altered epitope utilization caused by the route and adjuvant was reported for the *Helicobacter pylori* urease.^59^

Essentially all of the dominant epitopes occurred within conformationally stable segments of F1-V, as represented by a positive aggregate *z*-score (Figure 5). However, the dominant epitopes were not centered on the stable segments. Rather they appear on the edges of the stable segments, as represented by the epitopes at residues 65–80 in both C57BL/6 and CBA mice and at residues 275–290 in C57BL/6 mice. This offset from center of stability was the basis for the development of the antigen processing likelihood (APL) algorithm, which upweights the prediction on the edge of stable segments.^38^

**Figure 8:**
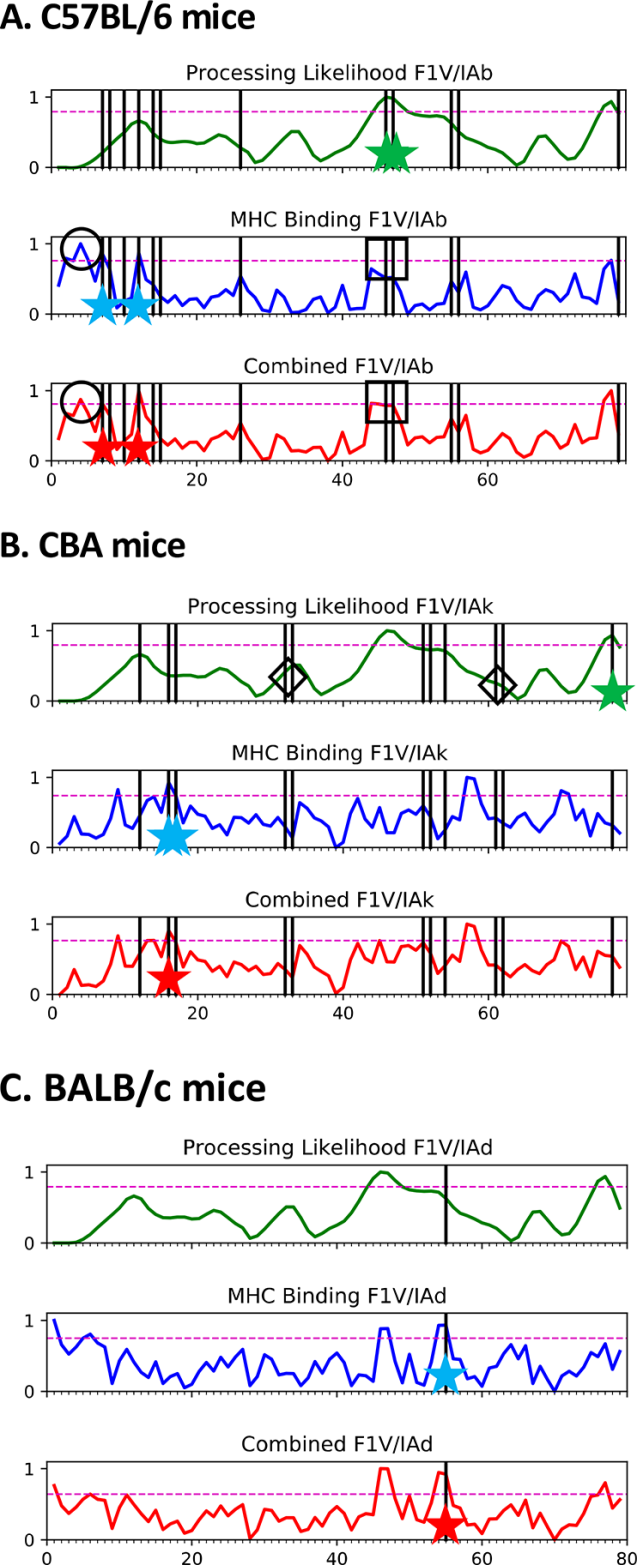
Combination of APL and MHCII binding for the prediction of dominant CD4+ epitopes of F1-V in (A) C57BL/6, (B) CBA, and (C) BALB/c mice. Dashed lines indicate the 90th percentile of prediction score. Vertical lines indicate the dominant epitopes identified by T-cell proliferation, and stars show correctly predicted epitopes by each method. Three of four false positives in the MHCII binding prediction (A, empty circles) for C57BL/6 mice were eliminated when combined with the APL prediction. APL also identifies two epitopes (A, squares) not identified by MHCII binding prediction. Some false negatives in the APL prediction might be corrected by improvement of the algorithm (B, empty diamonds).

Selected dominant epitopes reveal strengths and weaknesses of APL and MHCII binding for epitope prediction. An important strength of APL is its potential to eliminate false positives and false negatives from the MHCII(I-A^b^) binding profile, which can be obtained from NETMHCII.^4^ For a false-positive example, peptides 2–5 are predicted to be epitopes in C57BL/6 mice by MHCII(I-A^b^) binding (Figure 8A, circle). None is predicted by APL, and only peptide 4 is predicted in the combined profile. Although peptides 2–4 stimulated responses in four mice, none was dominant, and this can be explained by the peptides’ location within the unstable N-terminal segment (residues 19–35) of Caf1 (Figures 3 and 4). The modest immunogenicity of peptides 2–5 is most likely due to destructive processing, as reflected in the low APL scores. In contrast, the nearby dominant peptides 8 and 12 scored highly in both MHCII binding and APL. APL can also correct false negatives. For a false-negative example, peptides 46 and 47 were not predicted to be epitopes by MHCII(I-A^b^) binding (Figure 8A, square). In contrast, they are predicted to be epitopes by APL and were boosted by APL into the 89th percentile of the combined prediction.

An important weakness in APL may be represented by its failure to predict dominant epitopes in peptides 51, 52, 61, and 62 in CBA mice (Figure 8B, diamonds). Peptides 51 and 52 lie adjacent to the highly flexible and protease-sensitive fusion junction. Although we might expect these peptides to be well processed, they did not score in the 90th percentile of APL because this segment of F1-V is not as conformationally stable as other segments of the protein (Figure 5B, note the lower stability of residues 200–220 and 370–390, compared with other immunogenic segments). The APL algorithm assigns a score to residues according to conformational stability and upweights the score of residues adjacent to unstable segments in proportion to conformational stability at the residue receiving the score. Due to modest local stability, peptides 51, 52, 61, and 62 were not upweighted into the 90th percentile of APL score (Figure 8B). Likewise, peptides 61 and 62 were not upweighted into the 90th percentile even though they are on the N-terminal flank of loop-400.

Strategies for overcoming this weakness in APL scoring are under investigation. Remarkably, APL based solely on the biochemical analysis (F-LE + proteolysis) predicted both the 51–52 and 61–62 dominant epitopes (Figure 6). Thus, efforts to improve the prediction of proteolytic sensitivity from crystallographic data may also yield an improvement in epitope prediction.

For a collection of 13 antigens whose CD4+ T-cell epitopes have been systematically mapped in C57BL/6 mice, the combination of APL and MHCII(I-A^b^) surpassed either prediction method alone (Table 2). In each study, mice were primed with the antigen in a native-like state (e.g., not by peptides) and then probed by lymphocyte restimulation using a complete set of overlapping peptides spanning the antigen. The set contains no antigens of fewer than 200 amino acid residues and no more than one antigen of a homologous protein family (e.g., flavivirus envelope proteins). The size minimum accounts for the fact that small proteins tend to have reduced conformational stability and lack complex domain structure.^71^ Many proteins of fewer than 200 residues undergo two-state folding transitions.^72^ Thus, small proteins yield little complexity in fragmentation by limited proteolysis, and the structures have little effect on antigen processing and epitope dominance. For the collection of model antigens, an average of 10% of the tested peptides contained epitopes. As described in Experimental Section, we evaluated the positive predictive value for each antigen at a scoring threshold determined by the remaining antigens. In a real-life test, for example, this is comparable to choosing seven of 83 possible peptides from influenza hemagglutinin. For this protein, MHCII(I-A^b^) correctly scored one hit and the combination of MHCII(I-A^b^) with APL scored two hits (Table 2). For the 13 antigens, the average accuracy (using the antigen-specific thresholds) was 18% for APL, 27% for MHCII(I-A^b^) binding, and 36% for the combined prediction, and only the combined method achieved significant accuracy when the three methods were tested side by side (Figure 6A). The combined prediction also delivered at least one hit in all but two of the 13 antigens (Table 2).

For the combined prediction method on the C57BL/6 data set, each of the chosen sources of conformational stability contributes substantially to the optimal PPV results, with COREX and ASA having the highest weights. More generally, on all data sets tested we find that optimization of the input-source weights resulted in nontrivial weights for all input sources of conformational stability. Thus, the parameter values from our optimization demonstrate rigorously that the considered sources of conformational stability are essential for APL performance.

A natural question is how these optimized parameters change predictive performance when compared to our prior results. The current version of the APL algorithm and the benchmark data sets have been updated significantly from our initial effort,^38^ and thus, it is not possible to make a direct comparison to published results. However, we did compare the APL and combined prediction methods with previously chosen and currently optimized parameters. In our initial effort, we utilized equal weights for all input sources for the sake of simplicity and hand-selected algorithm parameters; in that work, we chose a magnification of 2, a flank size of 28, and a loop size of 0. Results for this comparison are shown in Figure S4. Overall, we find that the median PPVs are identical, but the optimized parameters do improve AUCs in all cases. The largest improvement in AUC was in the human data set, where manually selected parameters yield an AUC of 0.67 while the optimized parameters yield an AUC of 0.71. We can conclude that, while our prior parameter sets are reasonable, our optimization approach provides a stronger rationale for parameter selection and yields slightly improved performance.

APL could be a major asset for the prediction of CD4+ epitopes in human immune responses. In the original description of the algorithm, APL correctly identified epitopes at a rate of 23% of peptides in the 80th percentile for a set of nine systematically mapped antigens.^38^ Here, we have updated the set to eliminate antigens with fewer than 200 residues, included three new antigens, and replaced antigen-85A with its homologue, antigen-85B (Table 2). Antigen-85B was considered superior because its dominant epitopes were characterized by frequency in the cohort of human subjects, rather than average intensity, i.e., number of Elispots. For the updated set of antigens, the experimentally mapped epitopes occurred with a frequency of 17% within the series of overlapping peptides spanning the antigens (Table 3).

**Table 3:** Numbers of Peptides: Epitopes, Total, and Correctly Predicted for Human Subjects

antigen	no. of epitopes	ref	PDB entry	total no. of peptides	threshold (no. of peptides)	APL
Ad5 hexon	16	(60)	3TG7	134	0.17 (23)	8
*M.t.* Ag85b	9	(61)	1F0N	28	0.16 (4)	1
*M.l.* Hsp70	11	(62)	2V7Y^a^	49	0.16 (8)	2
NS3 helicase	7	(63)	1CU1	45	0.17 (7)	1
polio Vp1	5	(64)	1VBC	24	0.16 (3)	0
TBE Env	12	(65)	1URZ	120	0.18 (21)	2
tetanus toxoid	10	(66)	1Z7H	51	0.17 (8)	3
Ves v 5	7	(67)	1QNX	65	0.18 (11)	1
PE38-III	17	(44)	1IKQ	67	0.16 (10)	6
flu HA	6	(68)	2VIU, 1HTM	98	0.18 (17)	3
HIV GAG	6	(69)	4XFX^a^	22	0.16 (3)	1
*M.t.* Mal6G	2	(70)	6DNP	143	0.18 (26)	2
average	9.0			71	0.17 (11.75)	2.5

a Homology model template.

We evaluated PPV performance (Figure 6) with respect to an empirically determined threshold with the rationale that epitope frequency could be estimated from a given training set. However, in practice, we may wish to be more liberal or more stringent with our predictions. That is, we could optimize prediction by conducting parameter optimization at a particular chosen threshold (instead of the known epitope frequency) and then predicting at that same threshold. To study the effectiveness of this approach, we considered the trend of mean PPV performance at multiple thresholds for each data set. For the C57BL/6 data set, we tested the 80th, 85th, 90th, and 95th percentile thresholds and found a significant trend (*p* = 0.004) of increasing PPVs, which were 28%, 34%, 40%, and 51%, respectively. For data from human subjects, we found a similar trend, with PPVs of 29%, 30%, 31%, 37%, and 43% at the 80th, 83rd, 90th, 91st, and 95th percentile thresholds, respectively. This shows a clear trend of more accurate predictions with more stringent thresholds, primarily due to reductions in the numbers of false positives. In particular, a PPV of 43% is comparable to the best MHCII binding-based CD4+ epitope predictions so far reported for human epitope mapping data.^17,73^

An important limitation of APL is the requirement of a high-resolution three-dimensional structure, typically an X-ray crystal structure. In a survey of the protein universe, 70% of sequences can be at least partially modeled with an existing experimental structure.^74^ For the purposes of CD4+ epitope prediction, such homology-modeled structures are adequate. Moreover, many of the protein sequences that cannot be modeled are likely to be natively unfolded and therefore poorly immunogenic for CD4+ T cells because they are protease-sensitive.^24,58^ Thus, APL may prove to be more useful than the structure requirement might initially suggest.
