# Supplementary material for: CD4+ T-Cell Epitope Prediction by Combined Analysis of Antigen Conformational Flexibility and Peptide-MHCII Binding Affinity
Source: Biochemistry. 2022 Jul 14;61(15):1585–99. doi: 10.1021/acs.biochem.2c00237 (PMC9352311; doi:10.1021/acs.biochem.2c00237)

Supplementary Information for "CD4+ T-cell epitope prediction by combined analysis of antigen conformational flexibility and peptide-MHCII binding affinity" by Tysheena Charles[1], Daniel L. Moss[1], Pawan Bhat[1], Peyton W. Moore[1], Nicholas A. Kummer[1], Avik Bhattacharya[2], Ramgopal R. Mettu[2], and Samuel J. Landry[1]

From the [1]Department of Biochemistry and Molecular Biology, Tulane University School of Medicine, New Orleans, LA 70112; [2]Department of Computer Science, Tulane University, New Orleans, LA 70118

This supplement contains 1 table and 4 figures in a total of 6 pages.

Supplemental Table 1. Caf1 and LcrV peptide sequences and frequencies of antibody and T-cell response (number of mice). Highlighted cells indicate dominant CD4+ T-cell epitopes.

	Peptide	Sequence	C57BL/6	CBA	BALB/c	C57BL/6	CBA	BALB/c
			Serum Reactivity			Proliferative Response		
1	Caf1-1	MKKISSVIAIALFGTIA	3	8				
2	Caf1-2	VIAIALFGTIATANAAD				4		
3	Caf1-3	FGTIATANAADLTASTT	1	2		4		
4	Caf1-4	ANAADLTASTTATATLV	1		1	4		1
5	Caf1-5	TASTTATATLVEPARIT		1				2
6	Caf1-6	TATLVEPARITLTYKEG	2					
7	Caf1-7	PARITLTYKEGAPITIM	1			7		
8	Caf1-8	TYKEGAPITIMDNGNID		1		8		
9	Caf1-9	PITIMDNGNIDTELLVG		1				
10	Caf1-10	NGNIDTELLVGTLTLGG		2		8	1	
11	Caf1-11	ELLVGTLTLGGYKTGTT		2				
12	Caf1-12	LTLGGYKTGTTSTSVNF	2	2		10	6	
13	Caf1-13	KTGTTSTSVNFTDAAGD	1	4				
14	Caf1-14	TSVNFTDAAGDPMYLTF	1	5		10		
15	Caf1-15	DAAGDPMYLTFTSQDGN	2	3		7		1
16	Caf1-16	MYLTFTSQDGNNHQFTT		3	1		10	
17	Caf1-17	SQDGNNHQFTTKVIGKD	2	2		4	10	1
18	Caf1-18	HQFTTKVIGKDSRDFDI	10	9	6			
19	Caf1-19	VIGKDSRDFDISPKVNG	10	7	6			1
20	Caf1-20	RDFDISPKVNGENLVGD	5	6	2			
21	Caf1-21	PKVNGENLVGDDVVLAT	2	4	1			
22	Caf1-22	NLVGDDVVLATGSQDFF	1	3	1			
23	Caf1-23	VVLATGSQDFFVRSIGS		3	2		3	
24	Caf1-24	SQDFFVRSIGSKGGKLA		5	2		4	
25	Caf1-25	RSIGSKGGKLAAGKYTD		4	2			
26	Caf1-26	GGKLAAGKYTDAVTVTV		4	1	7		
27	Caf1-27	GKYTDAVTVTVSNQ	3	6	2			
28	LcrV-1	MIRAYEQNPQHFIEDLE	10	9	8			
29	LcrV-2	EQNPQHFIEDLEKVRVE	11	8	10			
30	LcrV-3	FIEDLEKVRVEQLTGHG	6	5	6			
31	LcrV-4	KVRVEQLTGHGSSVLEE	1					
32	LcrV-5	LTGHGSSVLEELVQLVK					8	
33	LcrV-6	SVLEELVQLVKDKNIDI	1	1			9	
34	LcrV-7	VQLVKDKNIDISIKYDP	1	6				
35	LcrV-8	KNIDISIKYDPRKDSEV	8	4	4		5	
36	LcrV-9	IKYDPRKDSEVFANRVI	8	5	7			
37	LcrV-10	KDSEVFANRVITDDIEL		1				
38	LcrV-11	ANRVITDDIELLKKILA	2	2				

	Peptide	Sequence	C57BL/6	CBA	BALB/c	C57BL/6	CBA	BALB/c
			Serum Reactivity			Proliferative Response		
39	LcrV-12	DDIELLKKILAYFLPED						
40	LcrV-13	KKILAYFLPEDAILKGG	2		1			
41	LcrV-14	FLPEDAILKGGHYDNQL	4	3	8			
42	LcrV-15	ILKGGHYDNQLQNGIKR	3	3	1			
43	LcrV-16	YDNQLQNGIKRVKEFLE	3		1			
44	LcrV-17	NGIKRVKEFLESSPNTQ						
45	LcrV-18	KEFLESSPNTQWELRAF		1				
46	LcrV-19	SPNTQWELRAFMAVMHF	3	10		9	1	4
47	LcrV-20	ELRAFMAVMHFSLTADR	4		1	9		
48	LcrV-21	AVMHFSLTADRIDDDIL						
49	LcrV-22	LTADRIDDDILKVIVDS	2					
50	LcrV-23	DDDILKVIVDSMNHHGD	2					
51	LcrV-24	VIVDSMNHHGDARSKLR			1		9	1
52	LcrV-25	NHHGDARSKLREELAEL			1		7	
53	LcrV-26	RSKLREELAELTAELKI			1		4	
54	LcrV-27	ELAELTAELKIYSVIQA		7			6	
55	LcrV-28	AELKIYSVIQAEINKHL	1			10		7
56	LcrV-29	SVIQAEINKHLSSSGTI	3	1	2	8		
57	LcrV-30	INKHLSSSGTINIHDKS	1					
58	LcrV-31	SSGTINIHDKSINLMDK	1			5		
59	LcrV-32	IHDKSINLMDKNLYGYT			4			
60	LcrV-33	NLMDKNLYGYTDEEIFK			1			
61	LcrV-34	LYGYTDEEIFKASAEYK	1	1	1		10	
62	LcrV-35	EEIFKASAEYKILEKMP					10	
63	LcrV-36	SAEYKILEKMPQTTIQV	1					
64	LcrV-37	LEKMPQTTIQVDGSEKK	1				1	
65	LcrV-38	TTIQVDGSEKKIVSIKD	7		1			
66	LcrV-39	GSEKKIVSIKDFLGSEN	2			2	3	
67	LcrV-40	VSIKDFLGSENKRTGAL			1	2		1
68	LcrV-41	LGSENKRTGALGNLKNS	1		1			2
69	LcrV-42	RTGALGNLKNSYSYNKD	1	1				2
70	LcrV-43	NLKNSYSYNKDNNELSH	4	9	3		3	
71	LcrV-44	SYNKDNNELSHFATTCS	11	9	7	3	2	1
72	LcrV-45	NELSHFATTCSDKSRPL	9	9	3	2	1	
73	LcrV-46	ATTCSDKSRPLNDLVSQ	1					1
74	LcrV-47	KSRPLNDLVSQKTTQLS	1					
75	LcrV-48	DLVSQKTTQLSDITSRF		1	1			2
76	LcrV-49	TTQLSDITSRFNSAIEA	1	1				1
77	LcrV-50	ITSRFNSAIEALNRFIQ		1			6	1
78	LcrV-51	SAIEALNRFIQKYDSVM	2	1		4	2	2
79	LcrV-52	NRFIQKYDSVMQRLLDD		3				

Figure S1. According to SDS-PAGE and Coomassie staining, the fragmentation pattern from limited proteolysis with proteinase K remains consistent at pH 7.6, pH 6.6, and pH 5.6, suggesting that the folded structure in F1-V resists proteolysis down to pH 5.6. Two "+" indicates doubling of proteinase K concentration from 0.25 µg/mL to 0.5 µg/mL.

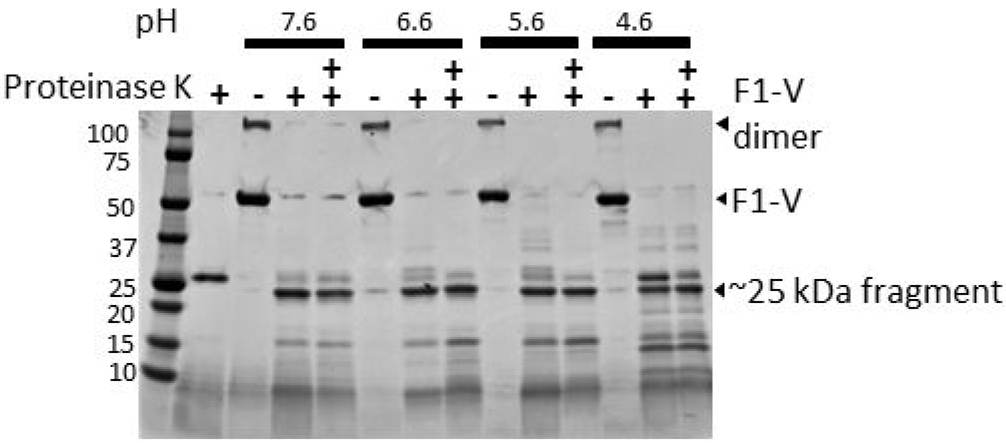

Figure S2. APL parameters for prediction of CD4+ epitopes optimized with a leave-one-out procedure (See Materials and Methods). In A, parameter distributions (median in red) for Combined MHCII binding and APL with thirteen antigens mapped in C57BL/6 mice. In B, parameter distributions (median in red) for APL only.

A. C57BL/6 Mice Combined

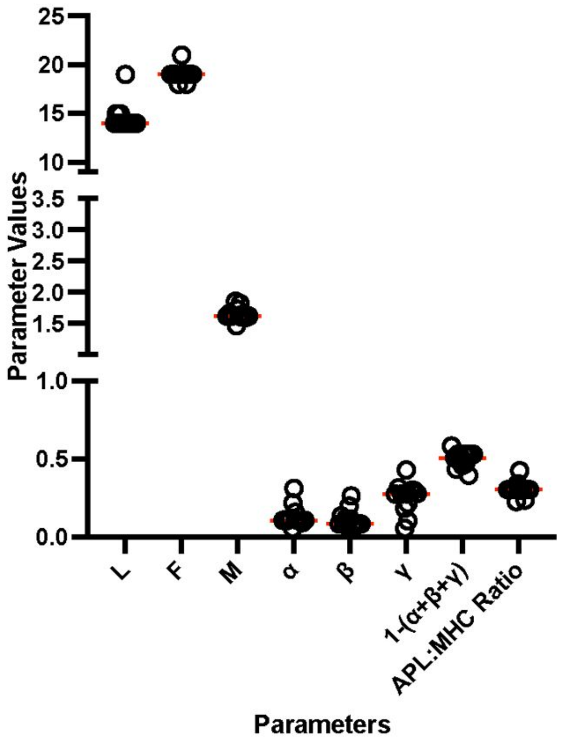

B. C57BL/6 Mice APL

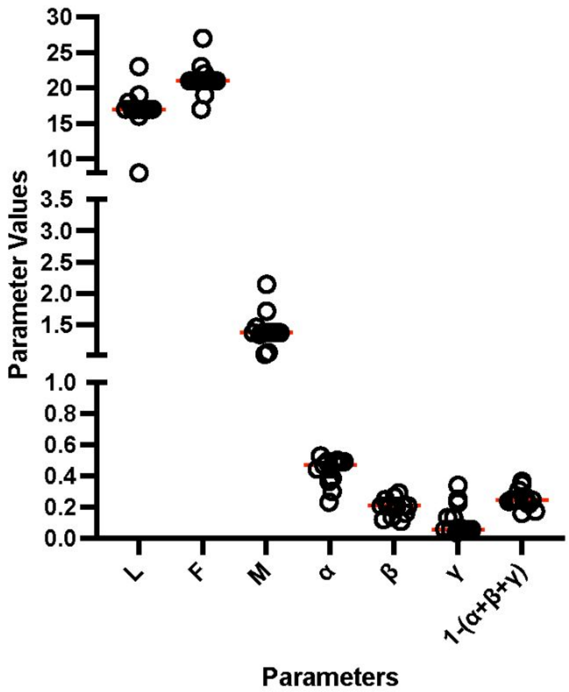

Figure S3. APL parameters distributions after optimization with a leave-one-out procedure (See Materials and Methods) for twelve antigens mapped in human subjects.

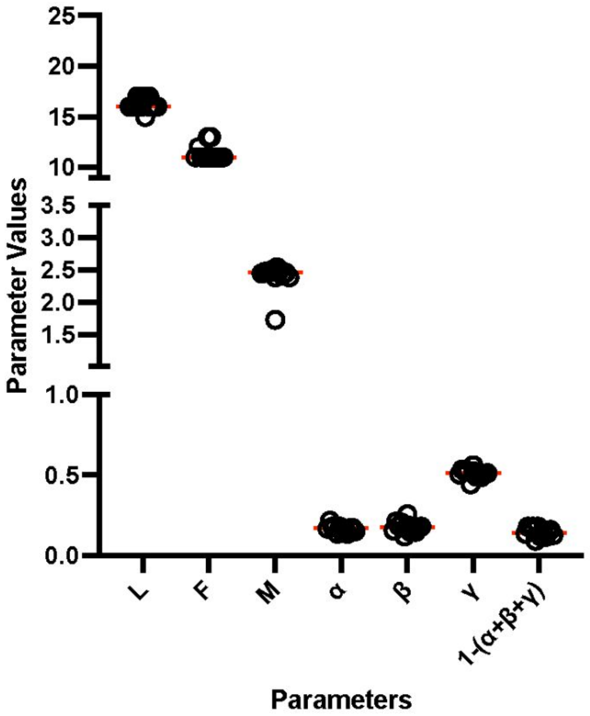

Figure S4. Comparison of prediction performance between optimized parameter sets and the parameter set used in our prior work (JIM 2016).

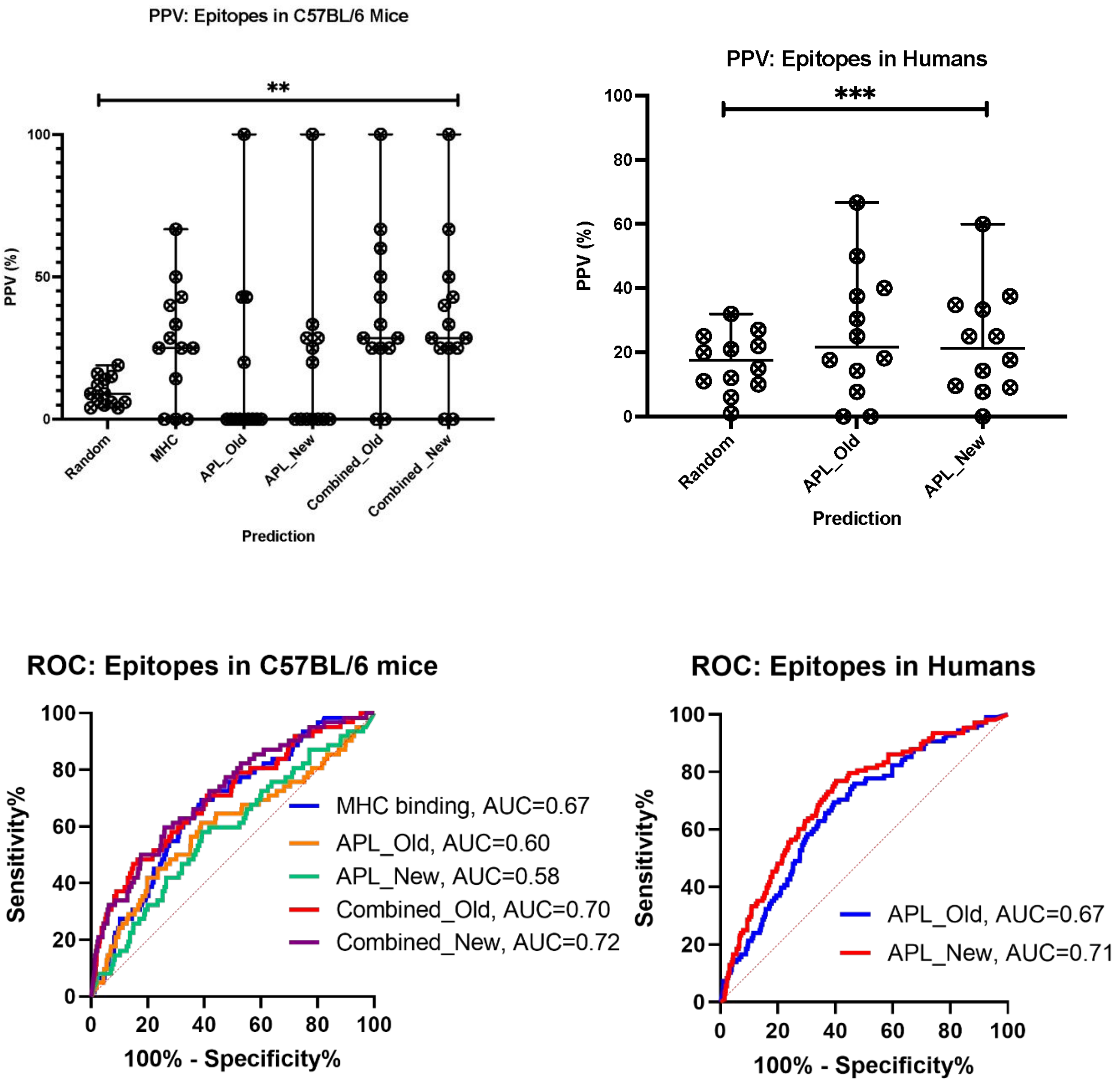

Supplement: Supplementary file 1 — bi2c00237_si_001.pdf [file bi2c00237_si_001.pdf]
